# Prognostic impact of ZAP-70 expression in chronic lymphocytic leukemia: mean fluorescence intensity T/B ratio versus percentage of positive cells

**DOI:** 10.1186/1479-5876-8-23

**Published:** 2010-03-08

**Authors:** Francesca M Rossi, Maria Ilaria Del Principe, Davide Rossi, Maria Irno Consalvo, Fabrizio Luciano, Antonella Zucchetto, Pietro Bulian, Riccardo Bomben, Michele Dal Bo, Marco Fangazio, Dania Benedetti, Massimo Degan, Gianluca Gaidano, Giovanni Del Poeta, Valter Gattei

**Affiliations:** 1 Clinical and Experimental Onco-Hematology Unit, Centro di Riferimento Oncologico, I.R.C.C.S., Aviano (PN), Italy; 2 Division of Hematology, S. Eugenio Hospital and University of Tor Vergata, Rome, Italy; 3 Division of Hematology - Department of Clinical and Experimental Medicine & BRMA - Amedeo Avogadro University of Eastern Piedmont, Novara, Italy

## Abstract

**Background:**

ZAP-70 is an independent negative prognostic marker in chronic lymphocytic leukemia (CLL). Usually, its expression is investigated by flow cytometric protocols in which the percentage of ZAP-70 positive CLL cells is determined in respect to isotypic control (ISO-method) or residual ZAP-70 positive T cells (T-method). These methods, however, beside suffering of an inherent subjectivity in their application, may give discordant results in some cases. The aim of this study was to assess the prognostic significance of these methods in comparison with another in which ZAP-70 expression was evaluated as a Mean-Fluorescence-Intensity Ratio between gated T and CLL cells (T/B Ratio-method).

**Methods:**

Cytometric files relative to ZAP-70 determination according to the three readouts were retrospectively reviewed on a cohort of 173 patients (test set), all with complete clinical and biological prognostic assessment and time-to-treatment (TTT) available. Findings were then validated in an independent cohort of 341 cases from a different institution (validation set).

**Results:**

The optimal prognostic cut-offs for ZAP-70 expression were selected at 11% (ISO-method) or 20% of positive cells (T-method), as well as at 3.0 (T/B Ratio-method) in the test set; these cut-offs yielded 66, 60 and 73 ZAP-70^+ ^cases, respectively. Univariate analyses resulted in a better separation of ZAP-70^+ ^vs. ZAP-70^- ^CLL patients utilizing the T/B Ratio, compared to T- or ISO-methods. In multivariate analyses which included the major clinical and biological prognostic markers for CLL, the prognostic impact of ZAP-70 appeared stronger when the T/B-Ratio method was applied. These findings were confirmed in the validation set, in which ZAP-70 expression, evaluated by the T- (cut-off = 20%) or T/B Ratio- (cut-off = 3.0) methods, yielded 180 or 127 ZAP-70^+ ^cases, respectively. ZAP-70^+ ^patients according to the T/B Ratio-method had shorter TTT, both if compared to ZAP-70^- ^CLL, and to cases classified ZAP-70^+ ^by the T-method only.

**Conclusions:**

We suggest to evaluate ZAP-70 expression in routine settings using the T/B Ratio-method, given the operator and laboratory independent feature of this approach. We propose the 3.0 T/B Ratio value as optimal cut-off to discriminate ZAP-70^+ ^(T/B Ratio less than 3.0) from ZAP-70^- ^(T/B Ratio more/equal than 3.0) cases.

## Background

The T cell specific zeta-associated protein 70 (ZAP-70), first identified by gene expression profiling of chronic lymphocytic leukemia (CLL) cells [1], has been the focus of many studies in the last few years, due to the ability of this molecule to act as an independent prognostic marker in CLL, when its expression is investigated by flow cytometry [2-5].

At least two approaches are currently employed to define ZAP-70 positivity in CLL by flow cytometry. The first approach is based on the signal obtained using an isotype-matched antibody as negative control [3,4] Accordingly, a CLL sample is defined as ZAP-70 positive when at least 20% of CLL cells have a signal exceeding that of isotypic control. The second approach is based on the expression of ZAP-70 in normal T cells, that constitutively express the protein and hence are utilized as an internal positive control. Following this strategy, a CLL sample is defined as ZAP-70 positive when at least 20% of CLL cells express ZAP-70 at levels comparable to those found in the residual T cell component [2,6] Given the different readouts utilized to define ZAP-70 positivity in CLL, it is not unexpected that a fraction of cases may result discordant when both approaches are applied to the same cohort of patients [7]. In particular, ZAP-70 expression intensity by T cells has been found to influence the evaluation of ZAP-70 positivity by CLL cells when the latter method is employed [6,7]. However, both approaches indistinctly suffer of an inherent variability, due to subjectivity in cursor placement to determine the percentage of ZAP-70 positive cells. To overcome the latter issue, subsequent reports suggested to evaluate ZAP-70 expression with methods relying upon evaluation of mean fluorescence intensity (MFI) values, as measured in the context of both CLL cells and residual normal B or T cells, rather than computing the percentage of positive cells [6,8-15]. Notably, these methods have been demonstrated to be more reproducible in multicenter comparisons, and more easily adaptable to thawed material [8,14,15].

In the present study, we used a test and validation strategy to evaluate the clinical impact of ZAP-70 expression, as determined by computing the ratio between MFI values separately obtained on T and CLL cells (T/B Ratio-method). As a test set, we took advantage of a consecutive series of 173 CLL cases, all with a complete clinical and biological prognostic assessment.

## Methods

### Patient characteristics and prognostic assessment

This study analyzed two separate cohorts of peripheral blood (PB) samples of untreated CLL patients overall accounting for 514 cases. Diagnosis of CLL was confirmed by morphology and cytometric immunophenotype, according to the recently published guidelines [16,17]. The first cohort (hereafter "test set") included 173 patients enrolled at the Division of Hematology, University of Eastern Piedmont, Novara. Samples were 79 females and 94 males, with a median age of 70 (range 42-91). A complete clinical and biological assessment was available for all samples, including Rai stage at diagnosis, β2-microglobulin, interphase fluorescence in situ hybridization (FISH) analysis, immunoglobulin heavy chain variable (IGHV) genes mutational status, and flow cytometric analysis of CD38 and CD49d expression. The second cohort (hereafter "validation set") included 341 patients enrolled at the Division of Hematology, S. Eugenio Hospital and University of Tor Vergata, Rome. These patients were 152 females and 189 males, with a median age of 65 (range 33-89).

Cytogenetic abnormalities were detected by standard interphase FISH carried out with locus-specific (on chromosomes 11, 13 and 17) or α-satellite DNA (on chromosome 12) Vysis probes (Abbott, London, UK) [18]. IGHV genes mutational status was analyzed as extensively described in previous reports by our groups [19,20] Flow cytometric analyses of CD38 and CD49d were done as previously described [18], using the cut-off point of 30% of positive cells for both markers [18,21-23]. Patients provided informed consent in accordance with local Internal Review Board requirements and Declaration of Helsinki.

### Flow cytometric analysis of ZAP-70 expression

All flow cytometric detections of ZAP-70 expression in PB samples belonging to the test set were performed at the Clinical and Experimental Onco-Hematology Unit of the Centro di Riferimento Oncologico (Aviano, Italy). Samples were either processed within 48 hours since collection (50 cases), or cryopreserved until analysis (123 cases). Cells were labeled with anti-CD19-APC, anti-CD5-PE-Cy7 and anti-CD3-PE-conjugated monoclonal antibodies (mAbs, Becton-Dickinson, San Jose, CA) for 20 minutes, then treated with fixing and permeabilizing reagents (Fix&Perm kit, Caltag, Burlingame, CA) according to the manufacturer's instructions, and finally stained with the Alexa-488-conjugated anti-ZAP-70 mAb (clone 1E7.2, Caltag). A second tube was prepared exactly as above, but substituting the Alexa-488-conjugated anti-ZAP-70 mAb with an isotype-matched Alexa-488-conjugated control mAb (Caltag). All samples were acquired on a FACSCanto flow cytometer and analyzed with DiVa software (Becton-Dickinson). No significant differences in term of ZAP-70 Mean Fluorescence Intensity (MFI) values were found by comparing fresh versus thawed samples, as judged by evaluating the T cell component (p = 0.14; see Additional file 1).

Flow cytometric detections of ZAP-70 in PB samples belonging to the validation set, all performed at the laboratory of the Hematology Unit, S. Eugenio Hospital, University of Tor Vergata (Rome, Italy), were an updating of previously reported analyses [22]. Briefly, PB mononuclear cells, separated on a density gradient (Ficoll-Hypaque, Pharmacia), were stained with anti-CD19-PerCP, anti-CD5-APC, anti-CD3/anti-CD56-PE mAbs, treated with the Fix&Perm kit (Caltag), and finally stained with the Alexa-488-conjugated anti-ZAP-70 mAb (clone 1E7.2, Caltag). Samples were acquired on a FACSCalibur flow cytometer and analyzed with CellQuest software (Becton-Dickinson).

**Figure 1:**
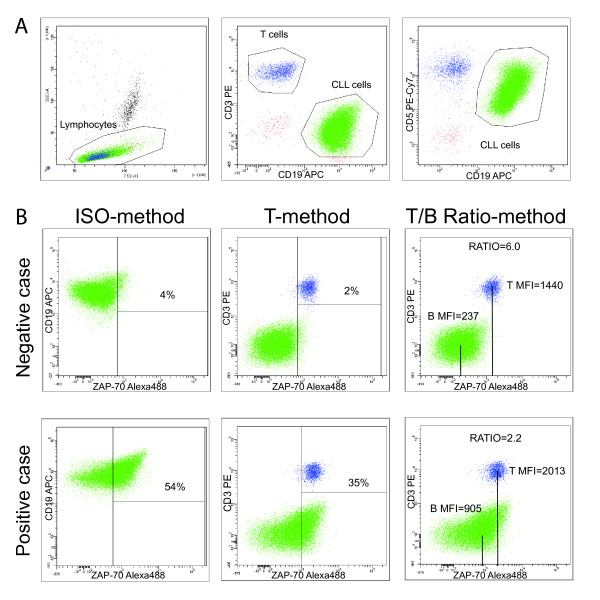
**Flow cytometric analysis of ZAP-70 expression (test set)**. PB cells of CLL samples were analyzed after staining with anti-CD19-APC, anti-CD3-PE, anti-CD5-PECy7 and AlexaFluor488-conjugated isotype control or anti-ZAP-70 antibodies. Panel A shows the gating strategies used to select lymphocytes in the left plot, CLL cells (CD19+/CD5+/CD3-) or T cells (CD19-/CD5+/CD3+) in middle and right plots, upon gating on lymphocytes. Panel B contains plots showing a representative ZAP-70 negative (upper row) and a representative ZAP-70 positive (lower row) sample, both analyzed according to the three different approaches utilized to evaluated ZAP-70 expression. The ISO- T-, and T/B Ratio-method readouts are shown respectively in the left, middle and right panels. For the ISO-method marker was set to have <1% CLL positive cells with isotypic control. For the T-method, marker was set on the left edge of T cells cluster, to have about 98% of positive cells. For the T/B Ratio-method the ratio was calculated directly from MFI values as separately read from T cell and CLL cell gates defined in panel A.

In all cases, at least 15 000 mononucleated cells and 2 000 T cells were acquired per tube. The lymphocyte population was gated based on morphological parameters on a forward- versus side-scatter (FSC/SSC) plot, excluding potential debris and lymphocyte doublets from the analysis. CLL and T cells were defined respectively as CD19^+^/CD5^+^/CD3^- ^or CD19^-^/CD5^+^/CD3^+ ^lymphocytes (Fig. 1A).

ZAP-70 expression was evaluated according to three different approaches (Figure 1B): i) a 2-tubes protocol, modified from the original protocol described by Rassenti et al. [4,7,24] (ISO-method); ii) a single-tube protocol, as originally described by Crespo et al. [2] (T-method); iii) a single-tube method calculating the ratio between the ZAP-70 Mean Fluorescence Intensity (MFI) values obtained from T and CLL cells (T/B Ratio-method).

According to the ISO-method (Fig. 1B, left panels), non-specific staining was evaluated on gated CLL cells in a CD19/isotypic control plot, setting the marker in order to have no more than 1% of positive cells (tube 1). This marker was then used to evaluate the percentage of ZAP-70 labeled CLL cells, as detected in tube 2.

The T-method (Fig. 1B, middle panels) implied the positioning of a marker close to the left edge of the T cell cluster in a ZAP-70/CD3 plot, and the use of this marker to calculate, in the same plot, the percentage of CLL positive cells. Although a skewed distribution of ZAP-70 in T cells was sometime observed [7], and considered in the positioning of the marker, this was usually set to have 98% of positive T cells.

The third approach (Fig. 1B, right panels) was based on the evaluation of ZAP-70 expression levels in terms of MFI, as measured on a CD3/ZAP-70 plot, utilizing the "mean" parameter, respectively on gated T lymphocytes (T-MFI), or CLL cells (B-MFI) as defined in plot A. These values were used to calculate the ratios between corresponding T-MFI and B-MFI (T/B Ratio-method).

### Statistical analysis

Statistical analyses were performed using the R statistical package with Design library [25]. Time-to-treatment (TTT) was measured from diagnosis to first line treatment, or last follow-up, and was available for all CLL cases entering the study. No deaths were recorded in the untreated patients or prior the start of therapy. Treatments were established following National Cancer Institute-Working Group guidelines [16]. The concordance index (c index) was used to determine the predictive ability of ZAP-70 positivity in a TTT model. Briefly, the c index is a probability of concordance between predicted and observed survival, with c = 0.5 for random predictions and c = 1 for a perfectly discriminating model [25]. An optimal cut-off for each of the three ZAP-70 readouts was chosen at the highest value of the c index, calculated for all the possible cut-off values of ZAP-70 [25]. TTT were estimated using Kaplan-Meier curves and comparison between groups were made by log-rank test. The Cox proportional hazard regression model was used to assess the independent effect of covariables, treated as dichotomous, on the TTT, with a backward procedure to select for significant variables. Coefficients of variation (CV) were calculated according to one way ANOVA test.

## Results and discussion

### ZAP-70 expression according to the ISO-, T- and T/B Ratio-methods

We first considered the cohort of 173 CLL patients included in the test set. Flow cytometric data files were re-analyzed according to the three different readouts applied to evaluate ZAP-70 expression (Fig. 1).

**Figure 2:**
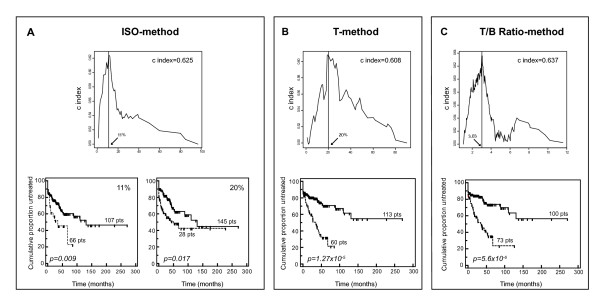
**C index and Kaplan-Meier curves for ZAP-70 evaluation according to ISO-, T- and T/B Ratio-methods (test set)**. Upper panels in A, B, and C show c index curves applied to ZAP-70 expression values to estimate the optimal cut-off capable to split patients into groups with different time to treatment (TTT) probabilities. X-axes report expression values for ZAP-70, expressed as percent of positive cells (A and B), or T/B ratio values (C); y-axes report the corresponding c index values. For each method, solid line indicates the chosen cut-off value. Lower panels show Kaplan-Meier curves obtained comparing TTT of patients affected by CLL expressing or not ZAP-70, as evaluated according to ISO- (A), T- (B) or T/B Ratio- (C) methods. In all plots, solid lines indicate ZAP-70 negative CLL, while dashed line indicate ZAP-70 positive CLL, according to the three readouts. In (A) Kaplan-Meier curves obtained by dividing CLL patients according to two different cut-offs (11% and 20%) for ZAP-70 evaluation are reported.

According to the ISO-method, in which ZAP-70 evaluation is driven by an isotypic control, 66/173 (38%) cases were defined as ZAP-70 positive using a cut-off value set at 11% of positive cells (Fig. 2A). This cut-off, in keeping with some pioneering studies on ZAP-70 expression and prognosis in CLL [3], was determined by selecting the value associated to the highest value of the c index. It was preferred to the standard 20% of positive cells, employed by other studies [4,24,26], which yielded in our series 28/173 ZAP-70 positive cases (16.2%), but a worse separation of ZAP-70+ vs. ZAP-70-cases (Fig. 2A). This result may be in part explained considering that CLL samples from the test set were analyzed either upon shipment by overnight courier or following thawing procedures, two conditions reported to potentially reduce ZAP-70 expression levels by CLL cells [14,27]. Consistently, a cut-off set at 15% of positive cells was also found to be more informative as a prognostic marker than the standard 20% in a series of frozen CLL samples retrospectively tested for ZAP-70 expression [27].

The T-method, in which ZAP-70 evaluation is driven by the residual population of normal T cells, yielded 60/173 positive cases (34.7%), by choosing the standard cut-off value of 20% positive cells to discriminate ZAP-70 positive vs. ZAP-70 negative CLL (Fig. 2B). At variance with the ISO-method, this cut-off was also associated with the best predictive ability as determined by the c index (Fig. 2B).

In the case of the T/B Ratio-method, in which ZAP-70 expression is evaluated taking into account T-MFI and B-MFI, the optimal cut-off value was again estimated by calculating the c index. As shown in Fig. 2C, a 3.0 T/B Ratio value was very near to the best cut-off selected for prognostic purposes. In our series, 100 CLL had T/B Ratio values greater or equal to 3.0 (i.e. ZAP-70 negative), while 73 CLL had values lower than 3.0, and were, therefore, considered as ZAP-70 positive cases (42.2%; Fig. 2C).

Approaches for evaluating ZAP-70 expression levels by computing the ratio between MFI values of CLL vs. T cells or T vs. CLL cells have been already proposed, although either applied to relatively small patient series, or without evaluating its prognostic relevance compared to the other methods currently employed in routine prognostic assessment of CLL patients [9-11,14,15,28]. Data presented here, suggesting a T/B Ratio value of 3.0 as the optimal cut-off point to discriminate ZAP-70 positive (i.e. with T/B Ratio values lower than 3.0) vs. ZAP-70 negative (i.e. with T/B Ratio values greater than or equal to 3.0) CLL, was obtained by utilizing the Alexa-488-conjugated 1E7.2 anti-ZAP-70 mAb. Although this mAb is one of the most frequently employed anti-ZAP-70 mAbs [4,5,24], several other mAbs have been reported, with different reactivity, fluorochrome conjugation, hence with different comparative performances [10,29]. Therefore, it would be not surprising that the 3.0 cut-off indicated by us could be influenced by the use of a particular anti-ZAP-70 mAb. As an example, a 4.5 was recently employed in a CLL series in which ZAP-70 expression was investigated by using the PE-conjugated SBZAP mAb [28]. Moreover, in a study by Le Garff-Tavernier et al. [14] a positivity threshold set at 4.0 was chosen by considering the mean value determined in a series of normal blood samples in which the ratio between expression of ZAP-70 in T vs. B cells was computed. Additional studied are therefore needed to validate the 3.0 cut-off, utilizing other anti-ZAP-70 clones and/or fluorochrome combinations.

In an attempt to evaluate the robustness of the T/B Ratio-method, as compared to the other approaches, ZAP-70 expression was independently evaluated by two operators (F.M.R. and A.Z.) in a series of 42 CLL. As reported in Additional file 2, although analyses were made by expert cytometrists, mean CV values computed for the three methods revealed a significantly higher variability when ZAP-70 expression was evaluated by the ISO-method (CV = 19.4) or the T-method (CV = 29.2) compared to the T/B Ratio-method (CV = 3.6). Accordingly, a technical report aimed at harmonizing different procedures for ZAP-70 evaluation among several laboratories, proposed an approach similar to our T/B Ratio-method as the method yielding the most accurate and reproducible results in both ZAP-70 positive and ZAP-70 negative cases [15].

### ZAP-70 expression according to the ISO-, T- and T/B Ratio-methods: prognostic significance

As summarized in Fig. 2, regardless of the readout chosen to evaluate ZAP-70 expression, high ZAP-70 levels always correlated with shorter TTT in CLL. This is in keeping with previous studies in which both ISO- and T-methods were proven to have prognostic relevance, also in wide cohorts of patients [5,24]. Nevertheless, a parallel comparison of the prognostic impact of different methods for ZAP-70 evaluation in a relatively wide CLL series is still lacking. In this regard, the Kaplan-Meier curves reported in Fig. 2 clearly showed that an evaluation of ZAP-70 expression utilizing the T/B Ratio-method yielded the best separation between ZAP-70 positive and ZAP-70 negative cases (p value = 5.6 × 10^-6^), followed by T- (p value = 1.3 × 10^-5^) and ISO- (p value = 0.009) methods.

This suggestion was confirmed by multivariate analyses, carried out in the whole series of 173 cases, in which ZAP-70 expression, as computed according to the three readouts, was included in a Cox proportional hazard regression model along with the main clinical and biological parameters (i.e. Rai stage, β2-microglobulin, FISH group, CD49d and CD38 expression, and IGHV gene mutational status) to test its relative strength as independent prognostic marker for TTT [18,30-33]. All the investigated parameters had prognostic impact by univariate analyses (Additional file 3). When included in a multivariate model, ZAP-70 expression, irrespective to the readout utilized, and FISH group were the sole biological parameters selected as independent prognostic markers along with the two clinical covariates (Table 1). Notably, regarding the prognostic impact of ZAP-70 expression in the three multivariate models, the highest value of hazard ratio (HR) was associated with the T/B Ratio-method, while lower HR values were found when ISO- or T-methods were considered (Table 1).

**Table 1:** Multivariate Cox regression analyses of TTT.

	HR (95% CI)*	p value
**Model 1 (ISO-method)**		
β_2_M (>2.2 g/L)	3.48 (1.73-7.03)	5.1 × 10^-4^
Rai stages (II-III-IV)	5.76 (3.56-9.33)	<1 × 10^-4^
FISH (+12,11q^-^,17p^-^)	1.76 (1.34-2.31)	5.6 × 10^-5^
ZAP-70 (≥ 11%)	2.11 (1.24-3.57)	5.7 × 10^-3^

**Model 2 (T-method)**		
β_2_M (>2.2 g/L)	3.16 (1.58-6.33)	1.2 × 10^-3^
Rai stages (II-III-IV)	5.97 (3.69-9.68)	<1 × 10^-4^
FISH (+12,11q^-^,17p^-^)	1.65 (1.26-2.17)	2.7 × 10^-4^
ZAP-70 (≥ 20%)	2.19 (1.29-3.72)	3.5 × 10^-3^

**Model 3 (T/B Ratio-method)**		
β_2_M (>2.2 g/L)	3.11 (1.55-6.23)	1.5 × 10^-3^
Rai stages (II-III-IV)	5.95 (3.65-9.71)	<1 × 10^-4^
FISH (+12,11q^-^,17p^-^)	1.64 (1.25-2.15)	4.1 × 10^-4^
ZAP-70 (<3.0)	2.72 (1.56-4.75)	4.5 × 10^-4^

Multivariate Cox regression analyses of TTT were performed on the 173 cases of the test set including the following covariates treated as dichotomous: β_2_-microglobulin (>2.2 g/L vs. ≤2.2 g/L); modified Rai staging (0-I vs. II-III-IV); FISH group (normal/13q^- ^vs. +12/11q^-^/17p^-^); CD38 (≥ 30% vs. <30%); CD49d (≥ 30% vs. <30%); IGHV mutational status (UM vs. M); and ZAP-70.

*Based on the final model after backward selection of covariates.

Abbreviations: TTT, Time-To-first-Treatment; HR, hazard ratio; CI, confidence interval.

### ZAP-70 expression according to ISO-, T- and T/B Ratio-methods: concordant and discordant cases

According to the three readouts examined, a percentage ranging from 34.7% (T-method) to 42.2% (T/B Ratio-method) of ZAP-70 positive cases was found. These values were lower than those reported by some literature studies, in which ZAP-70 positive cases were around or even exceeded 50% of CLL cases [24]. On the other hand, our results are in keeping with other studies investigating unselected, consecutive CLL series [34]. These differences can be explained considering the greater number of patients with low risk CLL usually enrolled by primary care centers. In the present series, 105/173 (66.5%) cases were classified as low-risk CLL by the modified Rai staging (Additional file 3), and 115/173 (60.7%) cases had a mutated IGHV gene status (see below). A similar proportion of ZAP-70 positive cases was found in other monocenter and multicenter Italian studies [5,18,19,35,36].

**Figure 3:**
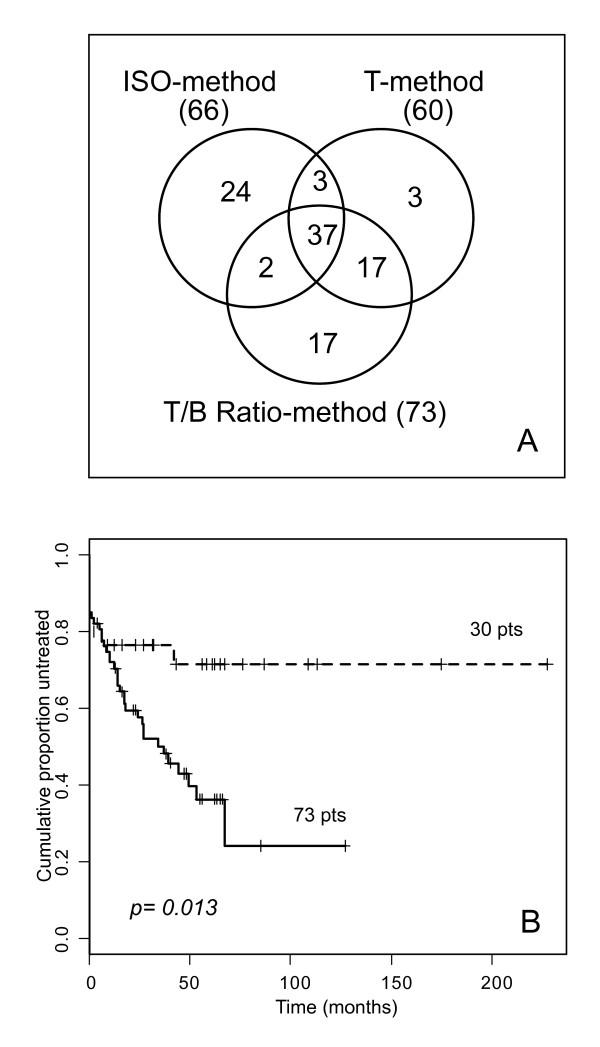
**Analysis of ZAP-70 concordant and discordant cases among ISO-, T- and T/B Ratio-methods (test set)**. (A) Venn diagram depicting concordant and discordant cases, as obtained by merging the ZAP-70 positive cases determined by ISO-, T- and T/B Ratio-methods. (B) Kaplan-Meyer curves obtained comparing TTT of patients affected by CLL expressing ZAP-70 according to T/B Ratio-method (73), or expressing ZAP-70 according to either ISO- or T-methods (30).

Overall, a total number of 103/173 cases (59.5%) turned out to be ZAP-70 positive utilizing at least one of the three readouts employed for ZAP-70 evaluation. These cases had a TTT significantly shorter than that of the remaining 70 cases, which were unequivocally negative for ZAP-70 expression, irrespective to the method employed for its evaluation (p = 0.001; Additional file 4). However, among these cases, only 37/103 were classified as ZAP-70 positive by all methods employed (i.e. concordant cases), while the remaining 66 CLL (discordant cases) were either ZAP-70 positive according to at least two methods (22 cases) or according to a single method (44 cases). A Venn diagram depicting concordant and discordant cases, as obtained by merging ZAP-70 positive cases according to the three readouts is reported in Fig. 3A. Notably, significantly shorter TTT intervals (p = 0.013) were observed in patients affected by ZAP-70 positive CLL according to the T/B Ratio-method (73 cases), compared to patients identified as ZAP-70 positive by the ISO- or the T-methods but not by the T/B Ratio-method (30 cases; Fig. 3B).

### ZAP-70 expression according to the ISO-, T- and T/B Ratio-methods: correlation with IGHV gene mutational status

IGHV gene mutational status represents an additional and commendable prognostic marker for CLL [20,21,37]. In the present series, 58/173 CLL had UM IGHV genes (33.5%). Again, this result is consistent with a consecutive CLL series without referral bias, and therefore relatively enriched in low risk cases [5,18,19,35,36]. As reported in Table 2, when IGHV gene mutational status and ZAP-70 positivity, determined according to the three readouts, were correlated, a significant concordance of 75%, 74% and 67% (p < 0.0001 for all readouts) was found by applying the ISO-, T- or the T/B Ratio-methods, respectively. This concordance rate is overall in keeping with other reports [2-5,24,38,39].

**Table 2:** Correlation of ZAP-70 analyses with IGHV mutational status as prognostic markers.

	**ISO-method**			**T-method**			**T/B Ratio-method**		
			
	**<11**	**≥ 11**	**% conc**	**<20**	**≥ 20**	**% conc**	**≥ 3**	**<3**	**% conc**
			
**M IGHV**	90	25	75	92	23	74	79	36	67
**UM IGHV**	17	41	(p < 0.00001)	21	37	(p < 0.00001)	21	37	(p < 0.00001)

Abbreviations: M IGHV, mutated IGHV genes status; UM IGHV, unmutated IGHV genes status; % conc, overall percentage of concordancy between the two prognostic parameters.

### Validation set: ZAP-70 expression by CLL and T cells

To validate the results obtained in the test set, we reviewed a different dataset of 341 CLL from another Institution, in which ZAP-70 staining and analyses were performed utilizing a different procedure and instrumentation. In this validation set, ZAP-70 expression was evaluated with the T-method utilizing the standard cut-off of 20% positive cells, as well as with the T/B Ratio-method; in the latter case, the cut-off of 3.0 identified in the test set was chosen.

**Figure 4:**
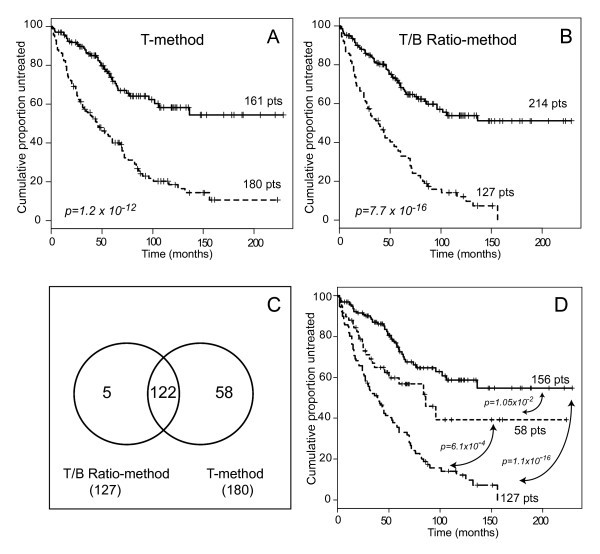
**ZAP-70 expression in the validation set**. (A-B) Kaplan-Meier curves obtained comparing TTT of patients affected by CLL expressing ZAP-70 according to T-method (A) or T/B Ratio-method (B). In all plots solid line indicates ZAP-70 negative CLL, while dashed line indicates ZAP-70 positive CLL. (C) Venn diagram depicting concordant and discordant cases, as obtained by merging the ZAP-70 positive cases determined by the two readouts. (D) Kaplan-Meyer curves obtained comparing TTT of patients affected by CLL expressing ZAP-70 according to T/B Ratio-method (127 cases), expressing ZAP-70 according to sole T-method (58 cases), or ZAP-70 negative according to both methods (156 cases).

According to the T-method, 180/341 cases (53%) were considered ZAP-70 positive, while when ZAP-70 expression was evaluated according to the T/B Ratio-method, the percentage of ZAP-70 positive cases decreased to 37.2% (127/341 cases). Again, a parallel comparison of the prognostic impact of the two methods for ZAP-70 evaluation clearly indicated a better separation between ZAP-70 positive and ZAP-70 negative cases when the T/B Ratio-method was applied (p value = 7.7 × 10^-16 ^vs. 1.2 × 10^-12^; Fig. 4AB).

As shown by the Venn diagram reported in Fig. 4C, 185 cases were overall classified as ZAP-70 positive by at least one procedure. Among them, 122 cases were concordantly positive, 58 cases were judged as ZAP-70 positive by the T-method only, while 5 cases were considered ZAP-70 positive solely by the T/B Ratio-method. Finally 156 cases were classified as ZAP-70 negative by both procedures. Notably, patients ZAP-70 positive according to the T/B Ratio-method (127 cases) experienced significantly shorter TTT intervals, both if compared to the 156 ZAP-70 negative cases, and to the 58 cases classified as ZAP-70 positive by the T-method only (Fig. 4D).

CLL samples belonging to the validation cohort were classified as positive for ZAP-70 expression according to data-defined criteria, as determined in the test set. Nevertheless, according to the c index curve computed also in the context of this dataset, we could confirm the 3.0 Ratio value for the T/B Ratio-method (actual value 3.15) as the optimal cut-off yielding the best segregation of ZAP-70 positive and ZAP-70 negative cases into two classes with different TTT probabilities (Additional file 5).

## Conclusions

In the present study, we had the opportunity to compare three different approaches for ZAP-70 evaluation in two separate cohorts of CLL patients, overall accounting for 514 cases. Notably, although in the two cohorts ZAP-70 was evaluated by utilizing the same antibody, two different mAb combinations, staining procedures and flow cytometers for data acquisition and analysis were employed. Despite this, the obtained results concordantly indicate that ZAP-70 expression, as evaluated by utilizing the T/B Ratio-method, appears to be a better predictor than the percentage of positive cells for progressive disease in CLL.

The underlying biological reasons explaining the stronger prognostic impact of ZAP-70 determination performed according to the T/B Ratio-method, compared to the other approaches based upon computation of percentages of positive cells, are still to be determined. In this regard, however, it has to be reminded that T/B Ratio values lower than the established 3.0 cut-off, as they are in CLL cases marked as ZAP-70 positive, can theoretically represent the result of a high expression level of ZAP-70 in the CLL component, but also of a low expression level of ZAP-70 by residual T cells. Previous studies by us and by other groups [6,7,40] documented highly heterogeneous levels of ZAP-70 by the residual T cell component of CLL samples. As an example, in the test set of the present study, MFI levels ranged from 370 to 3785. It is therefore tempting to speculate that peculiar biological features of the residual T cell component in CLL, as it could be identified by the variable expression of specific markers, e.g. CD38, telomeres, CD25 and CD54 [41-45] or, as shown here, ZAP-70, might be the result of interactions of T cells themselves with CLL cells, which might eventually contribute to define the clinical features of the disease [40,46].

The prognostic relevance of ZAP-70 determination in CLL has been emphasized in several retrospective analyses of wide cohorts of patients [5,24]. However, a standardized procedure for ZAP-70 evaluation, which allows to overcome the great interlaboratory variation associated with the different strategies and analytical approaches employed so far [47], although strongly recommended [48], is still lacking. Re-analyses of flow cytometric files by applying the T/B Ratio-method, as proposed here, could be useful for clarifying the real prognostic impact of this approach.

## Competing interests

The authors declare that they have no competing interests.

## Authors' contributions

Contribution: FMR wrote the manuscript, performed part of immunophenotypical studies and data analyses; MIDP and DR provided clinical data of patients and contributed to data analysis; RB, MDB. and MD performed the IGHV gene mutation and contributed to data analyses; AZ, DB, FL, and MIC performed part of immunophenotypical studies and contributed to data analysis; PB contributed to data analyses; M.F. provided clinical data of patients; GG provided patient samples and contributed to write the manuscript; GDP and VG coordinated the study and data analyses, and contributed to write the manuscript. All authors have read and approved the final manuscript.

## Supplementary Material

Additional file 1 **ZAP-70 expression in thawed vs. fresh samples**. Box and whiskers diagrams comparing the expression levels of ZAP-70, expressed as MFI values, in the T cell component of the 50 fresh vs. the 123 thawed CLL samples of the test set. Click here for file

Additional file 2 **ZAP-70 reading comparison between two different operators**. The table shows ZAP-70 expression levels calculated according to the ISO-, T-, and T/B Ratio-methods by two different operators on 42 cases belonging to the test set. Click here for file

Additional file 3 **Effect of the major clinical and biological prognosticators as TTT predictors in CLL from the test set**. Kaplan-Meier curves obtained comparing TTT of CLL patients split according to β2-microglobulin levels (A; >2.2 g/L vs. ≤ 2.2 g/L); modified Rai staging (B; low vs. intermediate vs. high risk); FISH groups (C; normal/13q^- ^vs. +12/11q^-^/17p^-^); IGHV gene mutational status (D; Mutated vs. Unmutated IGHV); CD49d (E; ≥ 30% vs. <30%); CD38 (F; ≥ 30% vs. <30%). Click here for file

Additional file 4 **Effect of ZAP-70 positivity as TTT predictor in CLL from the test set**. Kaplan-Meyer curves obtained comparing TTT of patients affected by CLL which were ZAP-70 positive (103) according to at least one readout (ISO-, T- and T/B Ratio-methods), or ZAP-70 negative (70) according to all readouts. Click here for file

Additional file 5 **C index curve for ZAP-70 evaluation in the validation set**. C index curve was used to estimate the optimal cut-off capable to split patients into groups with different time to treatment (TTT) probabilities applied to ZAP-70 expression values determined according to T/B Ratio-method. X-axis report expression values for ZAP-70, expressed as T/B ratio values; y-axis report the corresponding c index values. Click here for file
