# Supplementary figures and images for: Prognostic impact of ZAP-70 expression in chronic lymphocytic leukemia: mean fluorescence intensity T/B ratio versus percentage of positive cells
Source: J Transl Med. 2010 Mar 8;8:23. doi: 10.1186/1479-5876-8-23 (PMC2846891; doi:10.1186/1479-5876-8-23)

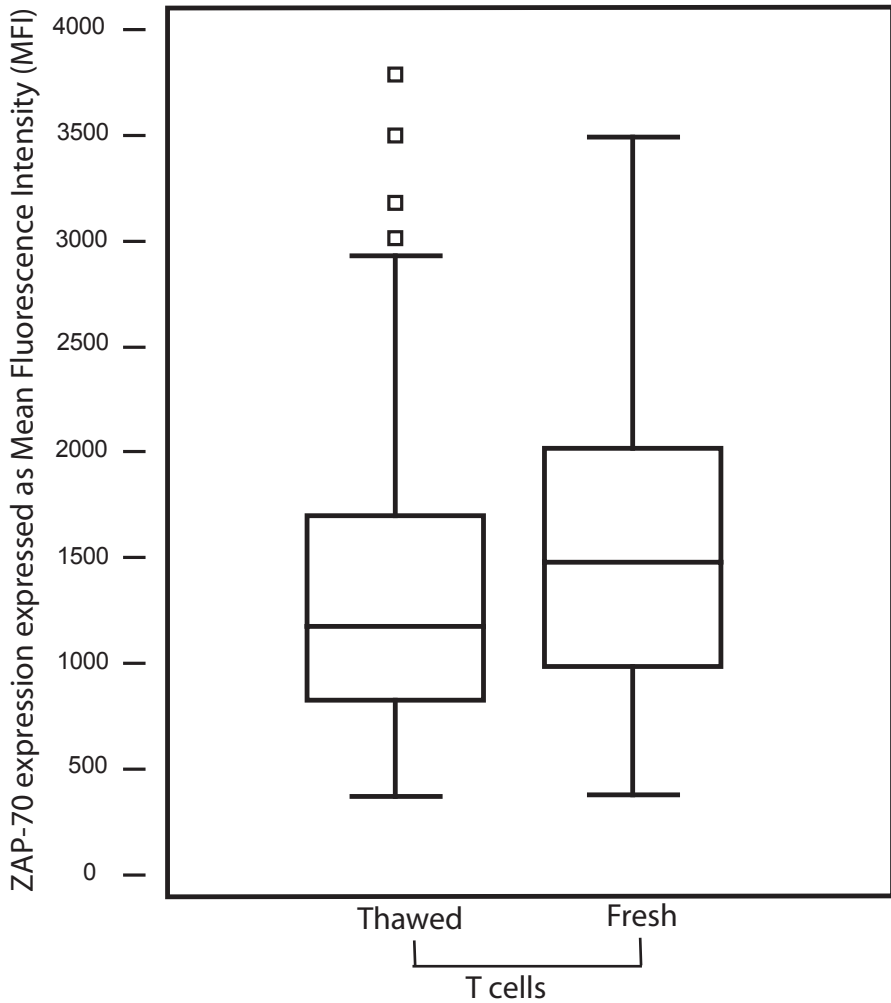

Supplement: Additional file 1 — ZAP-70 expression in thawed vs. fresh samples. Box and whiskers diagrams comparing the expression levels of ZAP-70, expressed as MFI values, in the T cell component of the 50 fresh vs. the 123 thawed CLL samples of the test set. [file 1479-5876-8-23-S1.PDF]

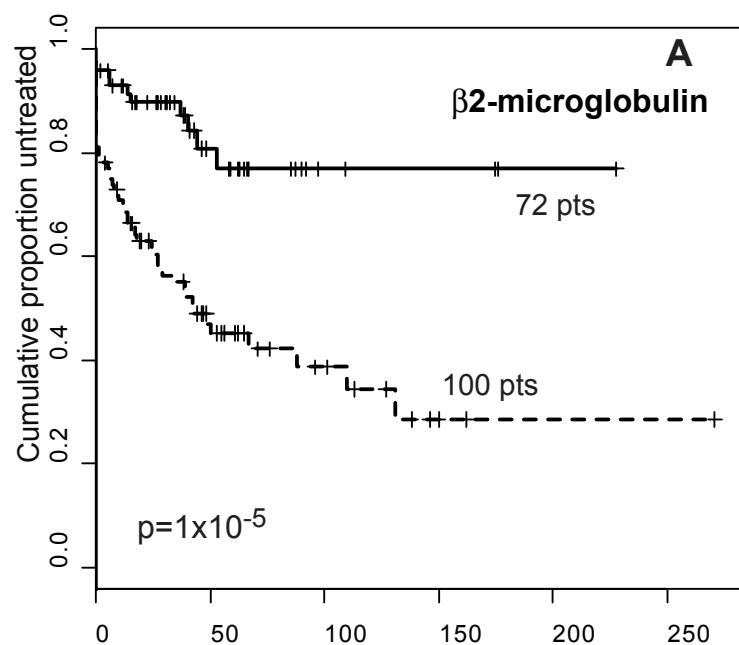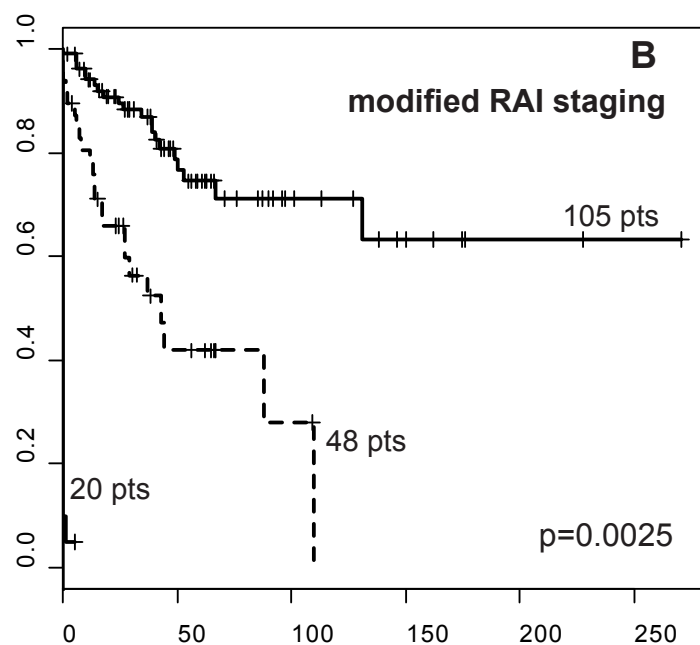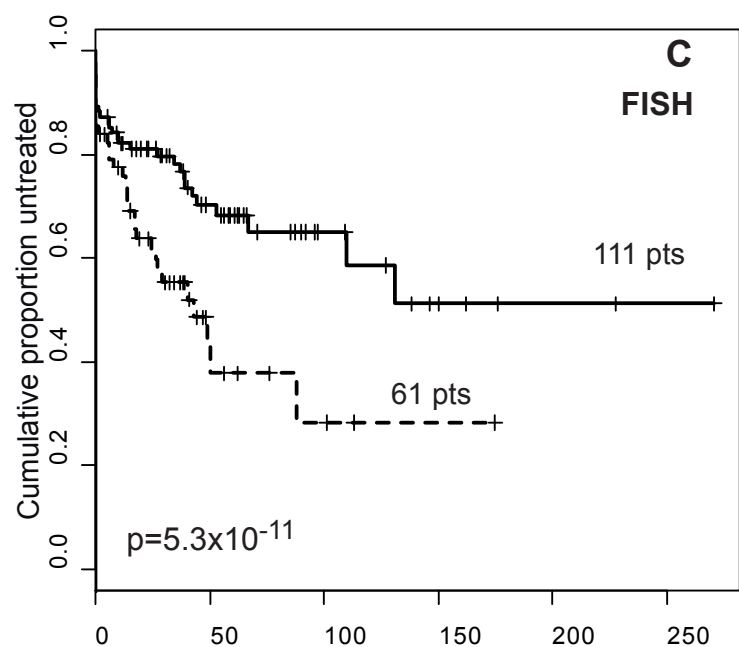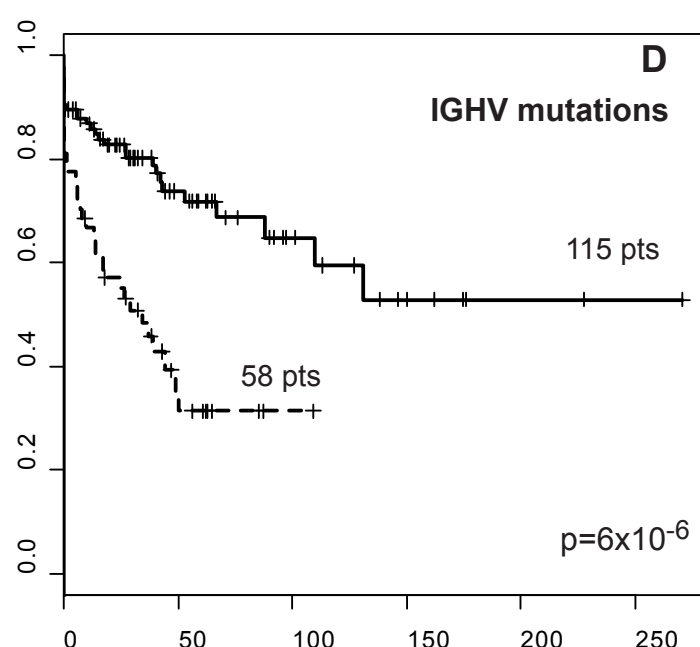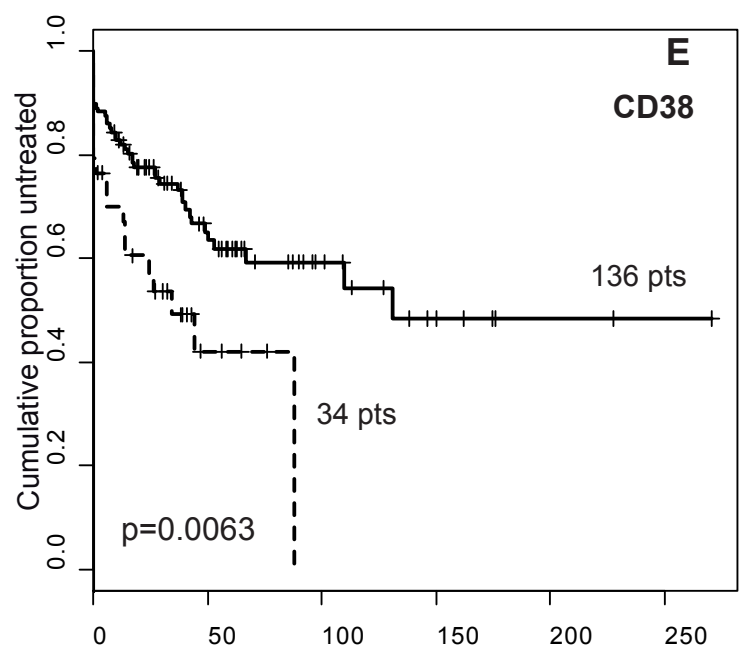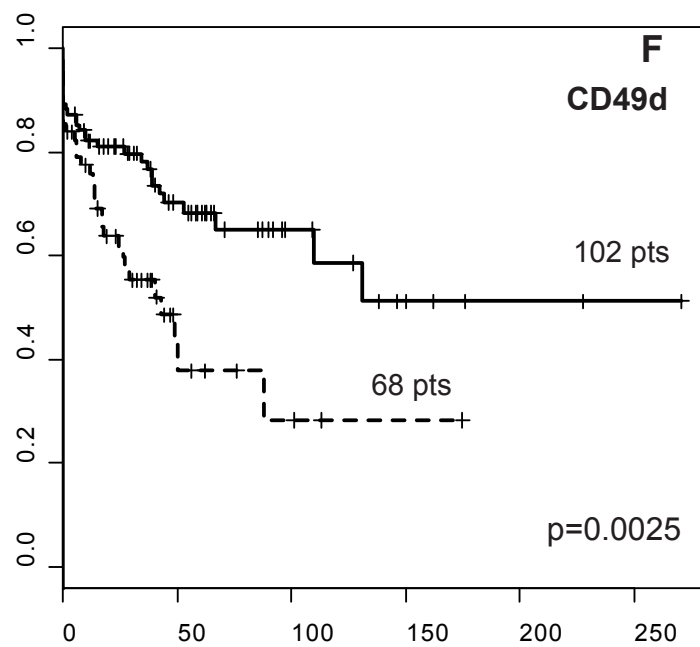

Supplement: Additional file 3 — Effect of the major clinical and biological prognosticators as TTT predictors in CLL from the test set. Kaplan-Meier curves obtained comparing TTT of CLL patients split according to β2-microglobulin levels (A; >2.2 g/L vs. ≤ 2.2 g/L); modified Rai staging (B; low vs. intermediate vs. high risk); FISH groups (C; normal/13q- vs. +12/11q-/17p-); IGHV gene mutational status (D; Mutated vs. Unmutated IGHV); CD49d (E; ≥ 30% vs. <30%); CD38 (F; ≥ 30% vs. <30%). [file 1479-5876-8-23-S3.PDF]

Cumulative proportion untreated

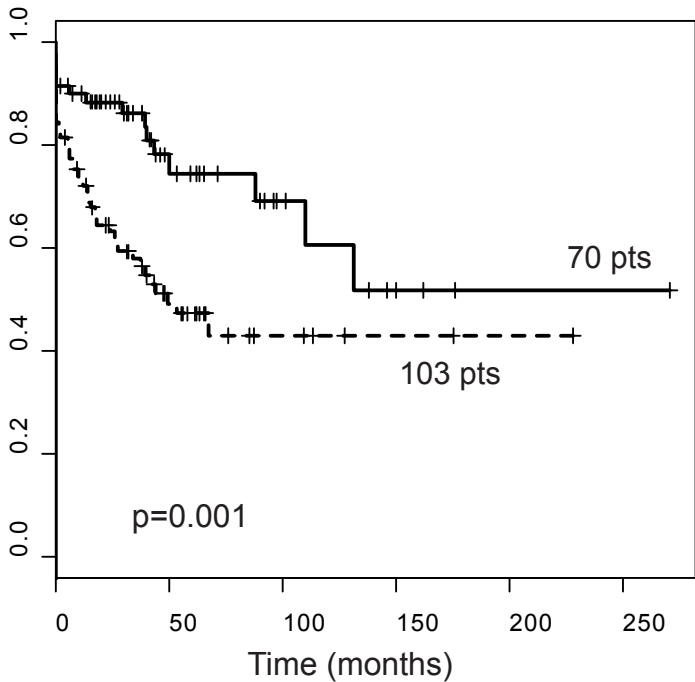

Supplement: Additional file 4 — Effect of ZAP-70 positivity as TTT predictor in CLL from the test set. Kaplan-Meyer curves obtained comparing TTT of patients affected by CLL which were ZAP-70 positive (103) according to at least one readout (ISO-, T- and T/B Ratio-methods), or ZAP-70 negative (70) according to all readouts. [file 1479-5876-8-23-S4.PDF]

# T/B Ratio-method

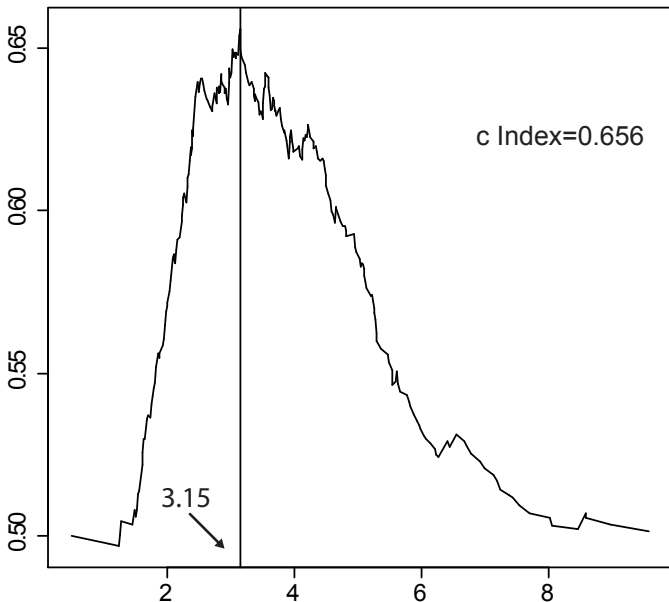

Supplement: Additional file 5 — C index curve for ZAP-70 evaluation in the validation set. C index curve was used to estimate the optimal cut-off capable to split patients into groups with different time to treatment (TTT) probabilities applied to ZAP-70 expression values determined according to T/B Ratio-method. X-axis report expression values for ZAP-70, expressed as T/B ratio values; y-axis report the corresponding c index values. [file 1479-5876-8-23-S5.PDF]
