# Supplementary material for: Prognostic impact of ZAP-70 expression in chronic lymphocytic leukemia: mean fluorescence intensity T/B ratio versus percentage of positive cells
Source: J Transl Med. 2010 Mar 8;8:23. doi: 10.1186/1479-5876-8-23 (PMC2846891; doi:10.1186/1479-5876-8-23)
Supplement: Additional file 2 — ZAP-70 reading comparison between two different operators. The table shows ZAP-70 expression levels calculated according to the ISO-, T-, and T/B Ratio-methods by two different operators on 42 cases belonging to the test set. [file 1479-5876-8-23-S2.PDF]

		I operator			II operator	
**Sample**	**ISO**	**T**	**T/B Ratio**	**ISO**	**T**	**T/B Ratio**
**1**	1.0	3.0	6.1	2.0	12.0	6.0
**2**	2.0	1.0	5.1	4.0	2.0	5.1
**3**	2.1	5.4	4.7	2.0	11.0	4.6
**4**	2.7	1.9	8.7	3.0	4.0	8.8
**5**	3.0	1.4	6.2	8.0	3.0	6.0
**6**	3.0	1.6	5.0	6.0	2.0	5.0
**7**	3.3	1.1	10.9	3.0	5.0	11.1
**8**	3.4	3.0	5.2	6.0	4.0	5.0
**9**	3.8	2.0	8.2	4.0	4.0	8.0
**10**	4.2	14.7	3.2	5.0	17.0	3.2
**11**	5.7	1.7	4.6	4.0	4.0	4.4
**12**	6.1	1.0	6.1	9.0	4.0	6.8
**13**	6.5	5.1	4.2	9.0	7.0	4.3
**14**	7.2	6.4	5.0	8.0	1.0	4.8
**15**	7.6	1.5	4.3	8.0	6.0	4.3
**16**	7.6	11.9	4.0	9.0	11.0	4.3
**17**	7.7	3.3	4.8	10.0	4.0	4.9
**18**	8.6	4.4	6.2	9.0	5.0	6.2
**19**	9.0	2.2	5.0	14.0	4.0	4.5
**20**	9.0	3.5	10.8	14.0	9.0	11.2
**21**	9.0	15.5	2.6	9.0	19.0	2.3
**22**	9.4	10.7	4.7	10.0	6.0	4.6
**23**	9.8	10.6	3.9	10.0	9.0	4.1
**24**	10.7	15.4	2.7	10.0	19.0	2.7
**25**	11.5	33.5	2.8	8.0	24.0	2.6
**26**	12.0	26.4	3.4	8.0	23.0	3.5
**27**	13.1	14.9	2.5	9.0	23.0	2.5
**28**	13.6	2.6	6.4	14.0	4.0	6.3
**29**	14.0	11.0	4.1	18.0	15.0	4.0
**30**	24.0	21.0	2.6	20.0	22.0	2.6
**31**	15.0	9.0	3.5	12.0	18.0	3.5
**32**	16.0	1.5	9.7	14.0	5.0	9.5
**33**	16.1	18.2	5.0	6.0	15.0	4.9
**34**	17.7	6.6	3.6	19.0	12.0	3.9
**35**	19.6	12.6	3.7	10.0	19.0	3.6
**36**	22.4	12.6	3.2	27.0	20.0	3.1
**37**	22.9	1.5	6.4	25.0	4.0	5.7
**38**	23.8	23.4	2.9	20.0	22.0	3.0
**39**	24.1	13.5	3.8	19.0	16.0	4.2
**40**	24.3	22.6	2.8	20.0	22.0	2.7
**41**	42.3	29.1	2.2	45.0	35.0	2.2
**42**	57.2	5.9	4.8	56.0	4.0	4.4
